# The early infant gut microbiome varies in association with a maternal high-fat diet

**DOI:** 10.1186/s13073-016-0330-z

**Published:** 2016-08-09

**Authors:** Derrick M. Chu, Kathleen M. Antony, Jun Ma, Amanda L. Prince, Lori Showalter, Michelle Moller, Kjersti M. Aagaard

**Affiliations:** 1 Department of Obstetrics and Gynecology, Baylor College of Medicine, Houston, TX USA; 2 Interdepartmental Program in Translational Biology and Molecular Medicine, Baylor College of Medicine, Houston, TX USA; 3 Medical Scientist Training Program, Baylor College of Medicine, Houston, TX USA; 4 Departments of Molecular & Human Genetics, Molecular & Cell Biology, and Molecular Physiology & Biophysics, Baylor College of Medicine, Houston, TX USA; 5 Division of Maternal-Fetal Medicine, Baylor College of Medicine, One Baylor Plaza, Jones 314, Houston, TX 77030 USA

**Keywords:** High-fat diet, Maternal gestational diet, Microbiome, Neonatal microbiome development

## Abstract

**Background:**

Emerging evidence suggests that the *in utero* environment is not sterile as once presumed. Work in the mouse demonstrated transmission of commensal bacteria from mother to fetus during gestation, though it is unclear what modulates this process. We have previously shown in the nonhuman primate that, independent of obesity, a maternal high-fat diet during gestation and lactation persistently shapes the juvenile gut microbiome. We therefore sought to interrogate in a population-based human longitudinal cohort whether a maternal high-fat diet similarly alters the neonatal and infant gut microbiome in early life.

**Methods:**

A representative cohort was prospectively enrolled either in the early third trimester or intrapartum (*n* = 163), with a subset consented to longitudinal sampling through the postpartum interval (*n* = 81). Multiple body site samples, including stool and meconium, were collected from neonates at delivery and by 6 weeks of age. A rapid dietary questionnaire was administered to estimate intake of fat, added sugars, and fiber over the past month (National Health and Examination Survey). DNA was extracted from each infant meconium/stool sample (MoBio) and subjected to 16S rRNA gene sequencing and analysis.

**Results:**

On average, the maternal dietary intake of fat ranged from 14.0 to 55.2 %, with an average intake of 33.1 % (σ = 6.1 %). Mothers whose diets significantly differed from the mean (±1 standard deviation) were separated into two distinct groups, a control group (*n* = 13, μ = 24.4 %) and a high-fat group (*n* = 13, μ = 43.1 %). Principal coordinate analysis revealed that the microbiome of the neonatal stool at birth (meconium) clustered differently by virtue of maternal gestational diet (PERMANOVA *p* = 0.001). LEfSe feature selection identified several taxa that discriminated the groups, with a notable relative depletion of *Bacteroides* in the neonates exposed to a maternal high-fat gestational diet (Student’s *t*-test, *p* < 0.05) that persisted to 6 weeks of age.

**Conclusions:**

Similar to the primate, independent of maternal body mass index, a maternal high-fat diet is associated with distinct changes in the neonatal gut microbiome at birth which persist through 4–6 weeks of age. Our findings underscore the importance of counseling pregnant mothers on macronutrient consumption during pregnancy and lactation.

**Electronic supplementary material:**

The online version of this article (doi:10.1186/s13073-016-0330-z) contains supplementary material, which is available to authorized users.

## Background

The human microbiome encompasses a rich ecosystem of approximately 90 trillion microbes that aid in human metabolism and impact host physiology [1, 2]. Next-generation sequencing technologies have allowed for more detailed characterization of the microbiome without the biases of culture-based techniques, enabling robust analyses linking microbiota to human disease [3]. To date, microbiota have been associated with obesity, inflammatory bowel disease, autoimmune disease, and, more recently, neurological disease related to the gut–brain axis [4–7]. Indeed, the interactions between a host and its microbiota are critical for proper developmental processes, but how host–microbe symbiosis is established and maintained across the lifespan remains underexplored.

Following gestation and birth, continued acquisition of a nascent microbiome in infancy is a relatively dynamic process thought to be primarily shaped by breastfeeding practices and weaning to solid food [8, 9]. Contrary to the long-held presumption that the *in utero* environment is sterile, emerging evidence has shown microbiota present in the placenta and amniotic fluid of healthy, term pregnancies devoid of clinical evidence of infection [10–14]. Moreover, in a murine model, orally administered bacteria have been cultured from the meconium of pups delivered by sterile cesarean, indicating that mother to fetus transmission of bacteria may occur during gestation [15]. Collectively, these findings suggest that colonization of the infant gut is likely to occur prior to parturition. Additional studies in well-controlled animal models are required to further characterize the mechanism of transmission.

Here, we sought to utilize a population-based, large, longitudinally sampled cohort of mother–infant dyads to interrogate the maternal factors that modulate microbial transmission of microbes in the peripartum period. In the adult, diet is a prominent modifier of the microbiome by providing selective metabolic pressures against or for specific microbes through substrate availability [16]. In a similar manner, we recently demonstrated in a non-human primate model that, independent of obesity, a maternal high-fat diet throughout gestation and lactation altered the composition of the offspring microbiome, which persisted up to one year of age regardless of the infant’s post-weaning diet [17]. These findings indicate that maternal diet in gestation, particularly fat intake and caloric density, may have a longstanding impact on the establishment and development of the infant microbiome. In the current study, we sought to determine if the effect of a maternal high-fat diet in gestation and lactation on the early infant microbiome could be recapitulated in a population-based prospective human cohort study.

## Methods

### Study design

The cohort data used in this study were part of a larger, population-based, prospective study that sought to characterize the origin and development of the neonatal microbiome across multiple body sites (skin, stool, oral cavity, nares). An initial cohort of 81 maternal–neonate dyads was prospectively enrolled in the third trimester from the clinical population at large. Power calculations based on the Dirilecht-multinomial distribution were done prior to subject enrollment with the anticipation of detecting a small effect size (ϕ = 0.07, α = 0.05) based on anticipated read counts (10,000 per sample) [18]. Mothers and infants were sampled at the time of delivery and at a follow-up visit 4–6 weeks later. Samples were collected across multiple body sites, representing the microbiome as a whole (stool, oral gingiva, nares, auricular and antecubital fossa skin, vaginal introitus, and posterior fornix). To increase the power of detecting a difference in the neonatal microbiome at delivery, a second, matched cross-sectional cohort (*n* = 82) was enrolled for sampling only at the time of delivery. Two mothers withdrew from the study and four delivered elsewhere and thus were excluded from the cohort. Detailed demographics of the two cohorts are provided in Additional file 1: Table S1. All subjects were enrolled and consented under Baylor College of Medicine Institutional Review Board H-27393. Enrollment criteria included gravidae 18 years or older with a viable pregnancy >28 weeks gestation. Subjects were excluded if there was known HIV or hepatitis C infection, immunosuppressive disease, use of cytokines or immunosuppressive agents within the past 6 months, a history of cancer except squamous or basal cell carcinoma of the skin managed by local excision, treatment of or suspicion of ever having had toxic shock syndrome, or major surgery of the gastrointestinal tract except cholecystectomy or appendectomy in the past 5 years.

### Maternal dietary estimation

To determine maternal dietary intake during pregnancy, each mother was asked by trained personnel at each sample collection time point to answer the Dietary Screener Questionnaire (DSQ), which was developed and validated by the National Health and Examination Survey (NHANES) program (2009–2010) [19, 20]. The DSQ is a rapid 26-question dietary screener that can be completed in a single visit and asks the respondent to provide the frequency of consumption of common foods. A copy of the DSQ is provided in Additional file 2. NHANES-provided scoring spreadsheets were used to convert frequency responses to daily intake estimates based on median portion sizes for reproductive-aged women (Additional file 3). A comparison of the dietary intake captured between the cohorts is shown in Additional file 1: Table S2 and the consistency of responses between time points from the same individual is given in Additional file 1: Table S3. To best recapitulate our non-human primate study, respondents reporting a dietary fat intake in the cohort extremes (±1 standard deviation from the cohort μ of 33.1 %) were divided into separate groups of identical sample size, representing a maternal gestational high-fat group (*n* = 13, μ = 43.1 %) and a control diet group (*n* = 13, μ = 24.4 %), which was used as reference for the high-fat cases (Additional file 4: Figure S1). Gestational weight gain was determined as previously described by comparing the weight gained in pregnancy for a given gestational age and pre-pregnancy body mass index (BMI) [21].

### Sample collection and processing

Neonatal meconium was obtained from the first diaper within 24–48 h of delivery. A second stool sample was collected from infants who were followed up at 4–6 weeks of age. Specimens were uniformly clean sterile collected by trained personnel according to a standardized protocol as previously described [3]. In a decontaminated, sterile environment, genomic DNA was isolated from each specimen using the MOBIO PowerSoil DNA Isolation Kit with the standard protocol. Extracted DNA was prepared for sequencing according to the HMP consortium outlined protocol [1] and subjected to 16S rRNA gene pyrosequencing using the V3-V5 primer set on the 454-FLX Titanium platform. Sequencing data were filtered, denoised, and processed on the QIIME platform (v1.9) [17]. Default parameters were used in picking operational taxonomic units (OTUs) to build the OTU table. A total of 2,690,000 high-quality reads were retained and mapped to 7838 unique OTUs.

### Statistical analysis

The significance of comparisons between continuous distributions was determined by an appropriate parametric or non-parametric test where appropriate. Measurements of beta-diversity were determined using principal coordinate analysis (PCoA) using unweighted UniFrac distances [22]. The significance of clustering was determined by PERMANOVA with 999 permutations. Heatmaps were generated in R using the pheatmap package. Hierarchical clustering was performed on Euclidean distances using complete linkage. Linear regression analysis and plotting were performed in PRISM. Linear effect size (LEfSe) analysis was performed using the default parameters to identify features that discriminated our dietary groups of interest [23].

## Results

### Study cohort and demographics

To interrogate the impact of maternal gestational diet on the neonatal gut microbiome, we examined stool samples collected from neonates enrolled in a larger, prospective, population-based study that sought to characterize the early neonatal microbiome across multiple body sites (gut, skin, oral cavity, nares). Mother–infant dyads (*n* = 157) were sampled at the time of delivery, with a subset (*n* = 75) consented for longitudinal sampling at 4–6 weeks postpartum. As shown in Additional file 1: Table S1, the cohort was comprised of primarily Hispanic women (85.4 %), who delivered singleton pregnancies (95.5 %) at term (38.4 weeks). The rate of cesarean deliveries (33.8 %) and preterm birth (10.8 %) were similar to the US national incidence and were not enriched for [24, 25]. For the purposes of this study, we focused our efforts on only the neonatal stool samples collected at the time of delivery (first meconium) and at 4–6 weeks of age. All samples were subjected to standard 16S rRNA gene sequencing and analysis.

### Assessment of maternal diet in pregnancy

In order to accurately assess maternal dietary intake during pregnancy, we employed the Dietary Screener Questionnaire (DSQ), which is a rapid dietary screener developed and validated by the National Health and Nutrition Examination Survey (NHANES) program [19, 20]. The DSQ is comprised of 26 questions that ask for the frequency of consumption in the past month of representative food and beverages. Based on the recorded responses, estimates of daily dietary intake of added sugars, fat, and fiber over the past month were determined, which, in our cohort, represented the maternal diet during the latter part of the third trimester.

**Fig. 1:**
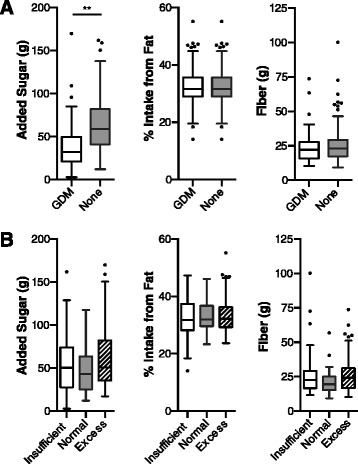
The maternal gestational diet is consistent with clinical expectations. Dietary intake of added sugars (g), fiber (g) and fat (percentage intake) during gestation compared **a** in mothers with or without gestational diabetes (*GDM*) or **b** in mothers with insufficient, normal, or excess gestational weight gain. Significance determined by Student’s *t*-test (**p = <0.01)

Comparisons between the captured dietary intake data from the study cohort, the average intake of reproductive-age women in the US, and the recommended daily dietary intake are shown in Table 1. In aggregate, the cohort’s average consumption of added sugars (59.6 ± 42 g) and total intake of fat (33.1 ± 6.1 %) was at or above the Institute of Medicine’s recommended limit, which is largely reflective of the poor quality diet in America. Notably, the average intake of added sugars in our study cohort was significantly less than the reported national average (59.6 ± 42 g versus 68.8 g, *p* = 0.01). However, this difference is likely explained by the fact that pregnant women who develop gestational diabetes are placed on a diabetic diet that limits carbohydrate intake; we had a significant proportion of gestational diabetics (30 %) in our study cohort [26]. As expected, women with gestational diabetes consumed significantly less added sugars (*p* < 0.001) but not fiber or fat (Fig. 1a). To further evaluate if the captured dietary intake values were consistent with clinical expectations, we similarly examined if dietary intake correlated with gestational weight gain. Although identifiable risk factors for gestational weight gain are widely variable between studies and thus poorly understood, previous studies have shown no association between dietary composition and gestational weight gain [27, 28]. Consistent with the literature, intake of added sugars, fiber, and fat did not significantly differ with gestational weight gain subject strata (Fig. 1b). In sum, the dietary intake data captured by the DSQ were similar to the average for reproductive-aged women in the US and generally consistent with clinical expectations.

**Table 1:** Comparison of maternal dietary intake in pregnancy

	Study cohort^a^ (*n* = 138)	US average^b^	Recommended^c^	Cohort versus US average^d^ (*p* value)
Fiber (g)	24.9 ± 13.2	15.8^e^	>25 g	0.001
Intake of fat (%)	33.1 ± 6.1	33.0^e^	20–35	0.92
Added sugar (g)	59.6 ± 42	68.8^f^	<25 g	0.01

^a^Values represent the mean and standard deviation of the cohort’s dietary intake per day. Dietary information was not captured for 19 mothers

^b^US average for reproductive-aged women (19–39 years) in the US reported

^c^Institute of Medicine recommendations [29]

^d^Significance determined by Student’s *t*-test

^e^“What We Eat in America”, NHANES 2011–2012 [59]

^f^Data from Ervin and Ogden [60]

### Impact of maternal fat intake during gestation on the neonatal microbiome

We previously demonstrated in a non-human primate model that, when compared with a control maternal diet (13 % fat), exposure to a maternal high-fat diet (36 % fat) during gestation and lactation persistently altered the offspring microbiome until up to one year of age [17]. To determine if this persistent effect of a maternal high-fat diet could be recapitulated in a human cohort, we modeled the conditions of our primate study by subdividing the study cohort into its extremes, defined as being one standard deviation greater or less than the cohort mean. Using this criterion, a high-fat maternal diet group (*n* = 13, 43.1 % fat intake) and maternal low-fat diet group (*n* = 13, 24.4 % fat intake) were identified for further analysis (Additional file 4: Figure S1). Notably, the average percentage dietary fat intake of the low-fat group fell within the recommended fat intake for the general population as indicated by the Institute of Medicine [29]; thus, for this analysis we considered this group as the control reference for the high-fat diet group comparisons and will refer to it as the “control group” from here on. As shown in Table 2, the percentage intake of fat significantly differed between the groups as expected (*p* < 0.001), while the intake of added sugar and fiber was not significantly different. Other potential confounders, such as pre-pregnancy BMI and mode of delivery, also did not significantly differ between the groups (all *p* > 0.05), though given the nested case-control design of this study, we may be underpowered to detect differences by these comparisons.

**Table 2:** Characteristics of groups segregated by extremes of maternal fat intake

	Control group^a^ (*n* = 13)	High-fat group^a^ (*n* = 13)	*p* ^b^
Intake of fat (%)	24.4 (18.4–26.8)	43.1 (39.3–55.2)^a^	<0.001
Added sugar	74.9 ± 37.4	50.9 ± 28.2	0.076
Fiber	33.1 ± 17.9	25.2 ± 11.8	0.197
Pre-pregnancy BMI	28.2 ± 7.7	24.2 ± 5.5	0.21
Gestational age	37.9 ± 3.9	38.0 ± 3.1	0.96
Gestational diabetes	1 (7.7 %)	4 (30.8 %)	0.32
Excess gestational weight gain	5 (38.5 %)	5 (38.5 %)	0.99
Preterm (delivery <37 weeks GA)	1 (7.7 %)	2 (15.4 %)	0.99
GBS positive	2 (15.4 %)	1 (7.7 %)	0.99
Cesarean delivery	3 (23 %)	3 (23 %)	0.99
Intrapartum antibiotics	7 (53.8 %)	6 (46.2 %)	0.99
Antepartum antibiotics	5 (38.5 %)	4 (30.8 %)	0.99
Chorioamnionitis	4 (30.8 %)	1 (7.7 %)	0.32
Neonate birth weight (percentile)	56.2 ± 25.4	62.5 ± 29.9	0.57
Infant gender (male, female)	7, 6	6, 7	0.99

^a^Values represent group average and range

^b^Significance determined by Student’s *t*-test or Fisher’s exact test where appropriate

*GA* gestational age

**Fig. 2:**
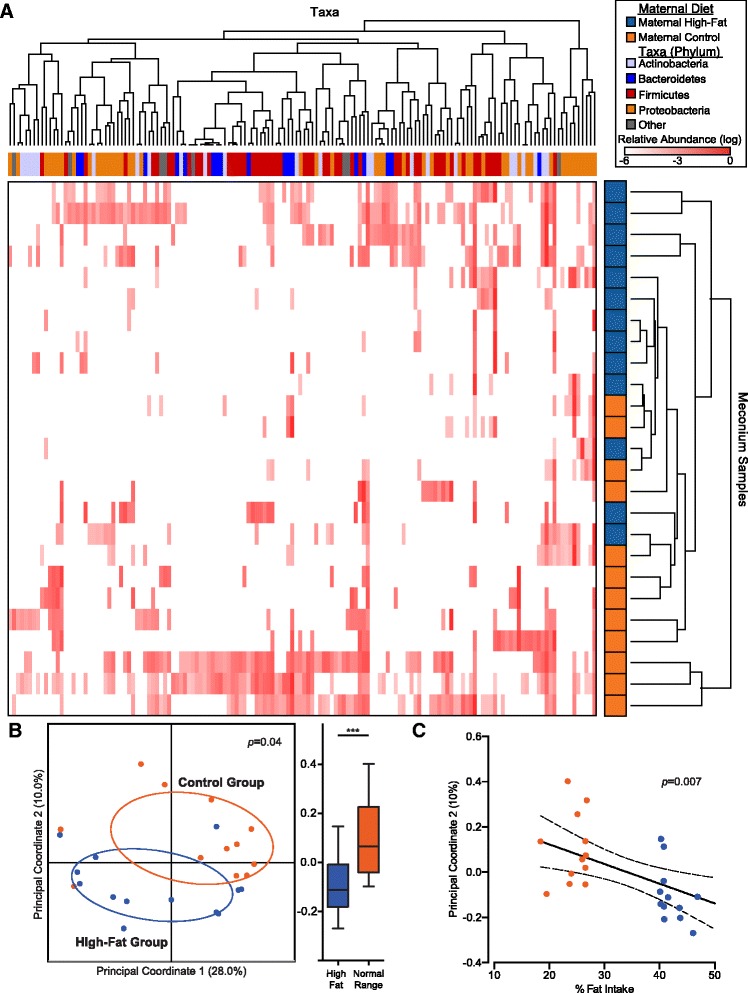
The neonatal gut microbiome at delivery varies according to maternal fat intake during pregnancy. **a** Heatmap showing unsupervised hierarchical clustering based on the relative abundance of each genera (*columns*) present in each meconium sample (*rows*). The maternal diet group (high-fat versus control) for each meconium sample and the phylum assignment for each genera are indicated by the *horizontal* and *vertical colored bars*, respectively. **b** Principal coordinate analysis of neonatal meconium on unweighted UniFrac distances, with the distribution of the samples along the second principal coordinate axis shown alongside on the *right* (***p < 0.001, Mann–Whitney U). **c** Linear regression between maternal gestational dietary fat intake and the second principal coordinate axis indicated by the *solid black line* with the 95 % confidence interval of the slope shown by the *dashed lines*

**Fig. 3:**
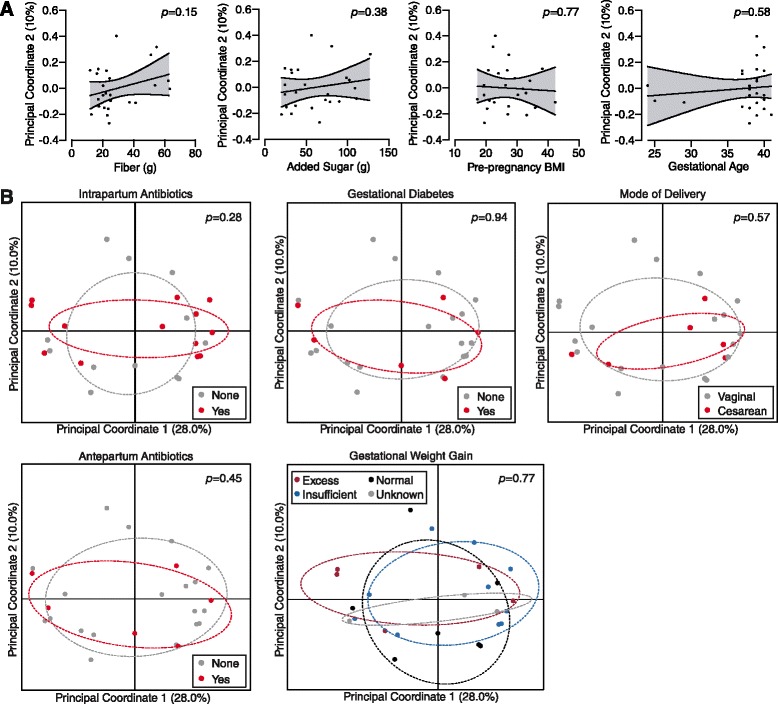
Variation of the neonatal gut microbiome is not explained by other potential confounders. **a** Linear regression between the second principal coordinate axis and maternal intake of fiber, added sugars, maternal pre-pregnancy BMI, and gestational age at delivery. All regression lines were not significantly different from 0, indicating no correlation. **b** Principal coordinate analysis of neonatal meconium, stratified by intrapartum antibiotic use, gestational diabetes, mode of delivery, antepartum antibiotic use, and gestational weight gain

To characterize and quantify the neonatal gut microbiome at the time of delivery, DNA from meconium samples was subjected to culture-independent 16S rRNA gene sequencing and analysis. With this approach, we identified 103 unique taxa classified down to at least the genus level that were represented in more than 10 % of all samples. To examine how maternal gestational diet correlated with the neonatal microbiome as a whole, we first projected the data as a heatmap, with each row representing the relative abundance of each taxa. Unsupervised hierarchical clustering on Euclidean distances revealed that a maternal high-fat diet in gestation varied in association with the neonatal microbiome (Fig. 2a). To further corroborate these findings, we next projected the data by principal coordinate analysis (PCoA) on unweighted UniFrac distances to reduce the dimensionality of the dataset to its components of greatest variation (principal coordinate (PC) axes 1 and 2). The neonatal microbiome again clustered significantly by virtue of maternal gestational diet (Fig. 2b; *p* = 0.04), which was best explained along the second PC axis (Fig. 2b, c). Notably, the variation of the neonatal microbiome along the second PC axis associated with maternal fat intake was not explained by maternal intake of fiber or added sugar (Fig. 3a; *p* > 0.05), nor was it confounded by other possible modifiers of the microbiome, including pre-pregnancy BMI, antibiotic usage, mode of delivery, gestational diabetes, or gestational weight gain (Fig. 3a, b; all *p* > 0.05).

**Fig. 4:**
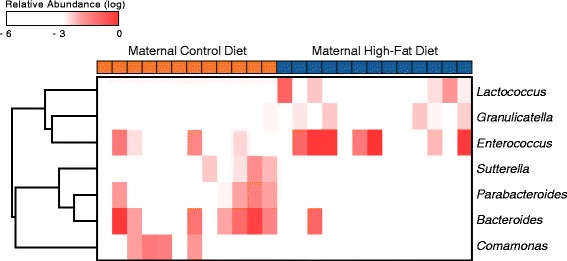
Specific taxa in the neonatal meconium significantly associated with maternal gestational diet. Heatmap of taxa in the neonatal meconium identified by LEfSe feature selection that were significantly associated with either a maternal high-fat or maternal control diet during pregnancy

We next employed linear effect size (LEfSe) feature selection [23] to identify the specific taxa in the neonatal microbiome that were significantly associated with either a maternal high-fat or control diet. Seven taxa were identified as significant and are shown in Fig. 4 as a heatmap indicating the relative abundance of each taxa in each neonatal meconium sample. Hierarchical clustering of the taxa indicated by the dendrogram along the vertical axis demonstrates the specific signature associated with either dietary group. Notably, exposure to a maternal high-fat diet was significantly associated with an enrichment of *Enterococcus* and a relative depletion of *Bacteroides*, which is a known symbiont that aids in the maturation of mucosal immunity (Fig. 4) [30–32].

**Fig. 5:**
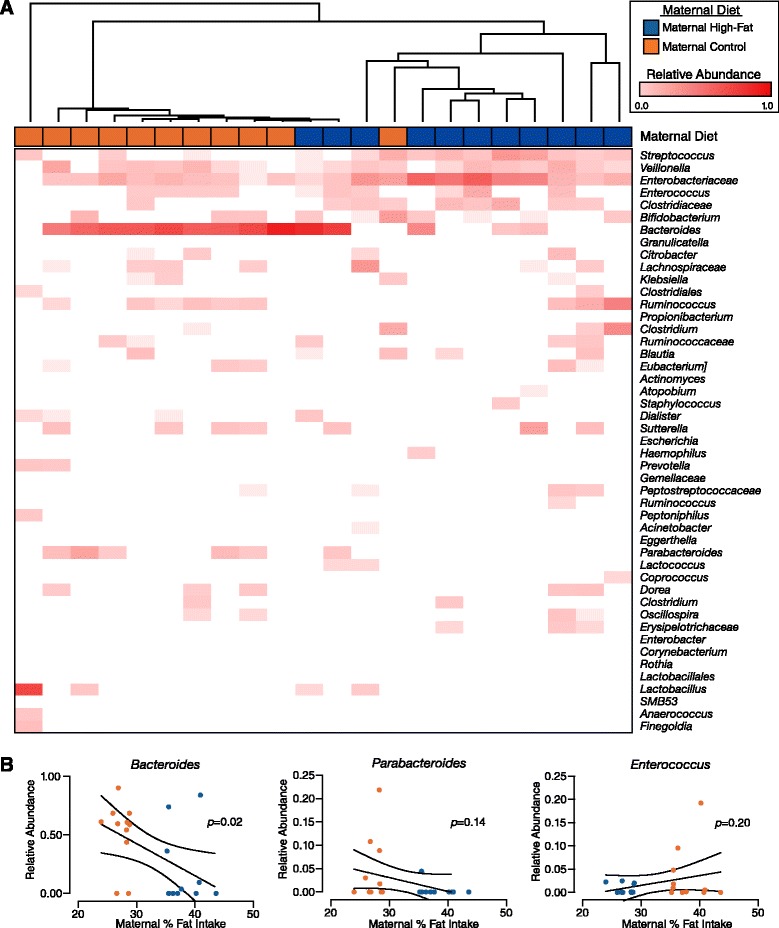
The impact of maternal gestational diet persists to 6 weeks of age. **a** Heatmap showing hierarchical clustering based on the relative abundance of the most abundant taxa in each infant stool sample, with the maternal diet group (high-fat versus control) indicated. **b** Linear regression between maternal gestational dietary intake and the relative abundance of *Bacteroides*, *Parabacteroides*, and *Enterococcus*

We next sought to determine if these changes in the neonatal gut microbiome associated with maternal gestational diet persisted beyond delivery. Similar to previous observations [8, 33], comparisons of the neonatal gut microbiome at delivery and at 6 weeks demonstrated significant differences at the phylum and OTU levels (Additional file 4: Figure S2), indicating broad rearrangements of the gut microbiome during this time period. When examining only the infant stool at 6 weeks, unsupervised hierarchical clustering again demonstrated significant clustering by maternal gestational diet (Fig. 5a), while clustering by unweighted PCoA trended toward significance (Additional file 4: Figure S3a; PERMANOVA *p* = 0.059). LEfSE analysis on the 6-week stool samples identified four taxa that were significantly associated with either the maternal high-fat or control diet group, though only *Bacteroides* was found to be significantly different at both delivery and 6 weeks (Additional file 4: Figure S3b). As in the neonatal meconium, the relative abundance of *Bacteroides* in the infant stool at 6 weeks of age was inversely correlated with the maternal fat intake during pregnancy (Fig. 5b; *p* = 0.02). Although the relative abundance of *Enterococcus* appeared subjectively higher in the infant stool exposed to a maternal high-fat diet in pregnancy, the correlation was not significant (Fig. 5c; *p* = 0.20). In addition, none of the other identified taxa at the time of delivery significantly correlated with maternal dietary intake (Additional file 4: Figure S4a; all *p >* 0.05). Breastfeeding practices have been shown to differentially impact the infant gut microbiome, with the greatest differences seen between those exclusively breast-fed or exclusively formula fed [33]. In our cohort, all infants received both breast milk and formula by the time they were sampled at 6 weeks of age, except for two in the high-fat diet group who were exclusively formula fed. When these two subjects were excluded from analysis, we still observed a significant correlation between the relative abundance of *Bacteroides* and maternal dietary fat intake during gestation (Additional file 4: Figure S4b; *p* = 0.04). Altogether, these findings indicate that a maternal high-fat gestational diet is significantly associated with specific alterations to the neonatal and infant gut microbiome, some of which persist to at least 6 weeks of age.

## Discussion

In this population-based prospective cohort study, we demonstrated that administration of a validated rapid dietary screener questionnaire adequately captured maternal dietary intake in the third trimester of pregnancy that was consistent with the US population and generally consistent with clinical expectations. Examination of the neonatal gut microbiome immediately after delivery revealed that it varied by virtue of maternal gestational diet, with a notable relative depletion of *Bacteroides* in the neonatal meconium exposed to a high-fat diet during gestation. At 6 weeks of age, the infant gut microbiome continued to vary by maternal gestational diet, with *Bacteroides* persistently depleted in infants exposed to a maternal high-fat diet in pregnancy.

In sum, the results from this study are in agreement with our previous published work in our non-human primate model [17]. In both cases, a maternal high-fat diet in gestation was associated with persistent and specific alterations in the offspring microbiome, though different taxa were shown to be altered in each case. Long standing exposure to a high-fat diet in the human population likely accounts for the differences seen between these studies. It is hypothesized that increased consumption of animal products over time has persistently altered the spectrum of commensal species native to the human gastrointestinal tract, which has been further indicated by the isolation of large amounts of *Helicobacter pylori* in the gut microbiome of a closer ancestral relative, Ötzi the Iceman [34, 35]. Therefore, perturbation of the human gut microbiome with a diet higher in fat may provide further selection pressures against commensal species like *Bacteroides*.

Persistent reduction of *Bacteroides* species in the infant gut could have significant consequences for efficient energy extraction and early immune development. *Bacteroides* species are major catabolizers of complex polysaccharides, including human milk oligosaccharides, which may be otherwise unusable by the host and other microbes [36, 37]. Fermentation of these polysaccharides into short chain fatty acids provides a major energy source for the host and is likely vitally important for the rapidly growing infant [36]. Additionally, polysaccharides generated by *Bacteroides* species promote healthy gut mucosal immunity by stimulating CD4 expansion and the production of the anti-inflammatory cytokine IL-10 [30–32, 38]. Many of these studies are based on the impact of one particular species, *Bacteroides fragilis*, which, although variably present in the neonatal gut in early life [39], has been shown to be transmitted from the maternal gut [40] and often is the dominant member of this important genus when present [41]. The resultant impact of maternal gestational diet on host immune development has been previously explored in murine and zebrafish models [42–44]. Interestingly, high-fat diet exposure during gestation and lactation had profound effects on offspring immunity and tolerance to inflammatory stimuli.

The relative depletion of *Bacteroides* species in this early time period may also influence an infant’s risk of developing obesity later on in life, but because the literature to date is limited, it is uncertain whether such an effect would be protective or contributory. Early studies observed that obesity in adults is associated with reduced levels of the phylum *Bacteroidetes,* which includes *Bacteroides* species [2, 45]; however, several more recent studies have seen opposite correlations whereby *Bacteroides* level are instead higher in overweight or obese subjects [46, 47]. The literature on the microbiota in overweight children is similarly limited and unclear, though several studies have observed increased *B. fragilis* in children with higher BMIs [48, 49]. The varied observations of previous studies likely reflects the number of confounding factors that are associated with obesity, including mode of delivery and diet, which may be difficult to control for and may alter data interpretations. For instance, obesity is an independent risk factor for cesarean delivery [50], which has been associated with delayed *Bacteroides* colonization in early life [33]. Additionally, obesity is a product of dietary intake, which can independently influence the abundance of *Bacteroides* [16]. Therefore, while the literature to date points to a potential role for *Bacteroides* species in modulating weight development in infants, additional well-controlled studies are needed to demonstrate causality rather than just correlation. Nevertheless, our reported data from the current study, as well as our prior analysis in a non-human primate model, renders the possibility that macronutrient intake may be equally important to the structure of the early neonatal microbiome, collectively underscoring the importance of maternal diet in pregnancy in shaping the health and well-being of the offspring.

The mechanism through which maternal gestational diet alters the offspring microbiome remains unclear. Alterations to the maternal microbiome associated with diet are thought to impact the bacterial transmission to the neonate, but when this occurs and which body sites matter most are uncertain. Identification of differences in the microbiome of the neonate’s first meconium indicates that the effect of maternal gestational diet may extend into the gestational interval and well before parturition occurs. The composition of the meconium is thought to originate from the large amounts of amniotic fluid swallowed by the infant and is thus thought to reflect the *in utero* environment [11]. Although it was presumed that the neonate was born sterile, an emerging body of work has begun to challenge this fundamental assumption. Recently, we characterized the placental microbiome using deep sequencing technologies, demonstrating that it appears most similar to the oral microbiome [21, 51]. Furthermore, Jiménez et al. [15] demonstrated that orally administered bacteria could be transferred from the pregnant dam to the fetal gut before delivery occurred. Findings from Callado and colleagues further show in a cohort of neonates delivered by elective cesarean section that the placenta, amniotic fluid, and neonatal meconium share many similar taxonomic features, suggesting microbial transfer *in utero* [13]. For these reasons, we speculate that a high-fat diet during gestation may impact the composition of the maternal oral or gut microbiome, thereby indirectly affecting the placental microbiome and subsequent seeding of the fetus. However, further well-controlled animal studies are needed to interrogate these potential mechanisms further.

Maternal diet in the postpartum period was not explored in this study but is nevertheless an important consideration. Human breast milk contains a low-biomass microbiome that has been previously hypothesized to be partially derived from the maternal gut [52–54]. If this is indeed the case, continued maternal consumption of a high-fat diet in the postpartum period could potentially impact the breast milk microbiome, further exacerbating dysbioses seen in the early gut microbiome if the infant is breastfed. Although previous studies have demonstrated that the macronutrient composition (e.g., fatty acids) of human milk is, to a certain degree, influenced by maternal diet, how diet impacts the microbial composition of human milk is unknown. Given the overall importance of breast milk to infant health and its potential role in microbial transmission, further studies are warranted to address these knowledge gaps.

The results of this study may be limited by the inherent limitations of using a rapid dietary screener. In exchange for a shorter questionnaire that could be reasonably completed in a single clinic visit (inclusive of the potential need for an interpreter), total caloric intake cannot be assessed and overall reliability of the screener is less than that of a more extensive questionnaire. However, the strength of this study, namely the large prospective population-based cohort design, ensured that a wide range of dietary intakes was captured such that the extremes of the population could be subsequently compared. Furthermore, particular attention was given to control for maternal obesity and gestational weight gain (Table 2, Fig. 3). In a landmark study by Turnbaugh et al. [7], body habitus was shown to be transferrable through the gut microbiome, implicating a potential microbiome of obesity. Work by Galley et al. [55], Collado et al. [56], and Mueller et al. [57] further demonstrated that maternal obesity as measured by pre-pregnancy BMI was associated with differences in the early infant gut microbiome. However, in these studies by others, maternal dietary intake of fat was not accounted for and likely represents a potential confounding factor. Therefore, in contrast with this viewpoint, our prior [17, 21] data along other’s published work further support the notion that diet, independent of obesity, has a role shaping the offspring microbiome [16, 34].

## Conclusions

We show that a maternal high-fat diet during gestation is independently associated with significant changes in the early neonatal microbiome that persist by 6 weeks of age. These findings have direct implications for refining dietary recommendations in pregnancy. Although clear guidelines have been established for total caloric and micronutrient intake, the recommendations for macronutrients (processed sugars, fats, fibers, and carbohydrates) are less clear in the absence of either pre-exiting or gestational diabetes [26, 58]. Our data show that significantly increased (>1 standard deviation) dietary intake of fat above the limit recommended by the Institute of Medicine (>35 %) has a lasting impact on the offspring microbiome [29]. Although obesity and gestational weight gain may be difficult for patients to address in the short term, establishing healthy dietary habits in pregnancy may be more readily achievable with significant long-term benefits for both mothers and their children.

## Abbreviations

BMI, body mass index; DSQ, Dietary Screener Questionnaire; NHANES, National Health and Nutrition Examination Survey; OTU, operational taxonomic unit; PC, principal coordinate; PCoA, principal coordinate analysis

## Additional files

Additional file 1: Supplemental Tables S1–S3. (DOCX 115 kb) Additional file 2: NHANES dietary questionnaire. (DOCX 85 kb) Additional file 3: NHANES dietary calculations. (XLSX 128 kb) Additional file 4: Supplemental Figures S1–S4. (PDF 296 kb)
