# Supplementary material for: The early infant gut microbiome varies in association with a maternal high-fat diet
Source: Genome Med. 2016 Aug 9;8:77. doi: 10.1186/s13073-016-0330-z (PMC4977686; doi:10.1186/s13073-016-0330-z)
Supplement: Additional file 1: — Supplemental Tables S1–S3. (DOCX 115 kb) [file 13073_2016_330_MOESM1_ESM.docx]

**Supplemental Table 1: Demographics**

| **Characteristic** | **All**  (n = 157) | **Longitudinal Cohort** (n = 75)^a^ | **Cross-Sectional Cohort** (n = 82)^a^ | ***p***^b^ |
| --- | --- | --- | --- | --- |
| Maternal Age | 30.0 ± 5.9 | 29.5 ± 5.4 | 30.4 ± 6.4 | 0.32 |
| Ethnicity |  |  |  | 0.51 |
| Hispanic (%) | 134 (85.4) | 66 (88) | 68 (82.9) |  |
| Non-Hispanic Caucasian (%) | 2 (1.3) | 0 (0) | 2 (2.4) |  |
| African American (%) | 13 (8.3) | 5 (5.3) | 8 (9.8) |  |
| Asian (%) | 8 (5.1) | 4 (5.3) | 4 (4.9) |  |
| Gravidity | 3.1 ± 1.6 | 3.3 ± 1.7 | 3.0 ± 1.6 | 0.30 |
| Parity | 1.7 ± 1.4 | 1.8 ± 1.5 | 1.7 ± 1.3 | 0.55 |
| Pre-Pregnancy BMI | 27.8 ± 5.9 | 28.9 ± 6.0 | 26.7 ± 5.7 | 0.017 |
| Gestational Diabetes (%) | 47 (29.9) | 33 (44.0) | 14 (17.1) | <0.001 |
| Preeclampsia (%) | 14 (8.9) | 8 (10.7) | 6 (7.3) | 0.58 |
| Chorioamnionitis (%) | 17 (10.8) | 8 (10.7) | 9 (11.0) | 0.99 |
| Urinary Tract Infection (%) | 27 (17.2) | 12 (16.0) | 15 (20.0) | 0.68 |
| GBS Positive (%) | 16 (10.2) | 5 (6.7) | 11 (13.4) | 0.19 |
| Intrapartum Antibiotics (%) | 59 (37.6) | 29 (38.7) | 40 (48.8) | 0.26 |
| Mode of Delivery |  |  |  | 0.40 |
| Vaginal (%) | 104 (66.2) | 53 (70.6) | 52 (63.4) |  |
| Cesarean (%) | 53 (33.8) | 22 (29.3) | 30 (36.6) |  |
| Gestational Age at Delivery | 38.4 ± 2.4 | 38.7 ± 1.5 | 38.0 ± 2.9 | 0.13 |
| Preterm Delivery (<37 weeks) (%) | 18 (11.5) | 6 (8) | 12 (14.6) | 0.22 |
| Twins (%) | 6 (3.8) | 2 (2.7) | 4 (4.8) | 0.68 |
| Infant Gender |  |  |  | 0.43 |
| Male (%) | 77 (47.2) | 39 (50.6) | 38 (44.2) |  |
| Female (%) | 86 (52.8) | 38 (49.4) | 48 (55.8) |  |
| Birth Weight (g) | 3266 ± 653.6 | 3329 ± 545.6 | 3209 ± 735.5 | 0.25 |
| ^a^Values represent cohort means ± standard deviation  ^b^Significance between Cohorts Determined by Student’s t-test or Fisher’s exact test | | | | |

**Supplemental Table 2: Dietary Intake by Study Cohort**

| **Characteristic** | **Longitudinal Cohort** (n = 55) | **Cross-Sectional Cohort** (n = 82) | ***p***^a^ |
| --- | --- | --- | --- |
| Added Sugar (g) | 49.2 ± 5.4 | 66.5 ± 47.4 | 0.017 |
| % Intake From Fat | 32.6 ± 4.1 | 33.5 ± 7.0 | 0.36 |
| Fiber (g) | 22.4 ± 10.7 | 26.4 ± 14.5 | 0.09 |
| ^a^Significance determined by Student’s t-test | | | |

| **Category** | **Delivery**  (n = 138) | **Postpartum**  (n = 58) | **Unpaired t-test** *(p)* | **Paired t-test** *(p)***^a^** | **Effectiveness of Pairing^b^** |
| --- | --- | --- | --- | --- | --- |
| Fiber (g) | 24.9 ± 13.2 | 25.4 ± 19.2 | 0.8 | 0.51 | R = 0.58  *p* < 0.001 |
| Energy From Fat (%) | 33.1 ± 6.1 | 31.6 ± 5.4 | 0.09 | 0.45 | R = 0.36  *p* = 0.008 |
| Added Sugar (g) | 59.6 ± 42 | 59.1 ± 30.4 | 0.93 | <0.001 | R = 0.71  *p* < 0.001 |
| ^a^Significance determined by paired t-test of responses between same individuals queried at delivery and 6 weeks postpartum.  ^b^Consistency of individual responses between delivery and postpartum determined by Wilcoxon Rank Sum Test. Values represent the significance (*p*) and correlation (R) between paired responses. | | | | | |

**Supplemental Table 3: Maternal Dietary Intake Between Time Points**
