# Supplementary material for: The early infant gut microbiome varies in association with a maternal high-fat diet
Source: Genome Med. 2016 Aug 9;8:77. doi: 10.1186/s13073-016-0330-z (PMC4977686; doi:10.1186/s13073-016-0330-z)
Supplement: Additional file 2: — NHANES dietary questionnaire. (DOCX 85 kb) [file 13073_2016_330_MOESM2_ESM.docx]

| Visit Number:  Visit 1  Visit 2  Visit 3 |
| --- |
| \| **Y N** \| \| --- \|   1. During the past month, did you eat about the same as your friends and neighbors?  If NO, specify difference:   \| _______________________________________________ \| \| --- \| |
| 2. Do you have any food allergies?   \| **Y N** \| \| --- \|   If YES, specify:   \| ________________________________________________ \| \| --- \| |
| 3. During the past month, did you follow a special diet?  Vegan  Vegetarian  Diabetic  DASH/low sodium diet  Gluten-free diet  Lactose intolerance  None  Other, specify:   \| _______________________________________________­ \| \| --- \| |
| \| **Y N** \| \| --- \|   4. During the past month, did you take any vitamins or supplements?  If YES, specify:  Prenatal Vitamin  Iron  Calcium  Folic Acid/Folate  Other, specify:   \| ________________________________________________ \| \| --- \| |
| 5. During the past month, how much of a priority was nutrition for you?  Not at all important  Somewhat important  Moderately important  Mostly important  Very important  Not applicable/don’t know |
| 6. During the past month, did you usually drink tap or bottled water?  Tap  Filtered Tap  Bottled  Other, specify: ______________________________________ |
| 7. How often do you eat out or buy prepared food?  Never  Sometimes  About half the time  Most of the time  All the time |

| 8. During the past month, how often did you eat hot or cold cereal?  Never 🡪 Go to question 9  1 time last month  2-3 times last month  1 time per week  2 times per week  3-4 times per week  5-6 times per week  1 time per day  2 or more times per day | 11. During the past month, how often did you drink **regular soda or pop** that contains sugar? Do **not** include diet soda.  Never  1 time last month  2-3 times last month  1 time per week  2 times per week  3-4 times per week  5-6 times per week  1 time per day  2-3 or more times per day  4-5 times per day  6 or more times per day |
| --- | --- |
| 8a. During the past month, what kind of cereal did you usually eat?   \| _______________________________________________________ \| \| --- \| | 12. During the past month, how often did you drink **100% pure fruit juices** such as orange, mango, apple, grape and pineapple juices? Do **not** include fruit-flavored drinks with added sugar or fruit juice you made at home and added sugar to.  Never  1 time last month  2-3 times last month  1 time per week  2 times per week  3-4 times per week  5-6 times per week  1 time per day  2-3 or more times per day  4-5 times per day  6 or more times per day |
| 8b. If there was another kind of cereal that you usually ate during the past month, what kind was it? *If none leave blank.*   \| _______________________________________________________ \| \| --- \| |  |
| 9. During the past month, how often did you have any **milk** (either to drink or on cereal)? Include regular milks, chocolate or other flavored milks, lactose-free milk, buttermilk. Please do **not** include soy milk or small amounts of milk in coffee or tea.  Never 🡪 Go to question 11  1 time last month  2-3 times last month  1 time per week  2 times per week  3-4 times per week  5-6 times per week  1 time per day  2-3 or more times per day  4-5 times per day  6 or more times per day  10. During the past month, what kind of milk did you usually drink?  Whole or regular milk  2% fat or reduced-fat milk  1%, ½%, or low-fat milk  Fat-free, skim or nonfat milk  Soy milk  Other kind of milk – *print milk*   \| _______________________________________________________ \| \| --- \| |  |
|  | 13. During the past month, how often did you drink coffee or tea that had **sugar** or **honey** added to it? Include coffee or tea you sweetened yourself and presweetened tea and coffee drinks such as Arizona Iced Tea and Frappuccino. Do **not** include artificially sweetened coffee or diet tea.  Never  1 time last month  2-3 times last month  1 time per week  2 times per week  3-4 times per week  5-6 times per week  1 time per day  2-3 or more times per day  4-5 times per day  6 or more times per day |
| 14. During the past month, how often did you drink **sweetened** fruit drinks, sports or energy drinks, such as Kool-Aid, lemonade, Hi-C, cranberry drink, Gatorade, Red Bull or Vitamin Water? Include fruit juices you made at home and added sugar to. Do **not** include diet drinks or artificially sweetened drinks.  Never  1 time last month  2-3 times last month  1 time per week  2 times per week  3-4 times per week  5-6 times per week  1 time per day  2-3 or more times per day  4-5 times per day  6 or more times per day | 17. During the past month, how often did you eat any kind of **fried potatoes**, including french fries, home fries, or hash brown potatoes?  Never  1 time last month  2-3 times last month  1 time per week  2 times per week  3-4 times per week  5-6 times per week  1 time per day  2 or more times per day |
| 15. During the past month, how often did you eat **fruit**? Include fresh, frozen or canned fruit. Do **not** include juices.  Never  1 time last month  2-3 times last month  1 time per week  2 times per week  3-4 times per week  5-6 times per week  1 time per day  2 or more times per day | 18. During the past month, how often did you eat any **other kind of potatoes**, such as baked, boiled, mashed potatoes, sweet potatoes, or potato salad?  Never  1 time last month  2-3 times last month  1 time per week  2 times per week  3-4 times per week  5-6 times per week  1 time per day  2 or more times per day |
| 16. During the past month, how often did you eat a green leafy or lettuce **salad**, with or without other vegetables?  Never  1 time last month  2-3 times last month  1 time per week  2 times per week  3-4 times per week  5-6 times per week  1 time per day  2 or more times per day | 19. During the past month, how often did you eat refried beans, baked beans, beans in soup, pork and beans, or any other type of cooked dried beans? Do **not** include green beans.  Never  1 time last month  2-3 times last month  1 time per week  2 times per week  3-4 times per week  5-6 times per week  1 time per day  2 or more times per day |
| 20. During the past month, how often did you eat **brown rice** or other cooked whole grains, such as bulgar, cracked wheat, or millet? Do **not** include white rice.  Never  1 time last month  2-3 times last month  1 time per week  2 times per week  3-4 times per week  5-6 times per week  1 time per day  2 or more times per day | 23. During the past month, how often did you eat **pizza**? Include frozen pizza, fast food pizza, and homemade pizza.  Never  1 time last month  2-3 times last month  1 time per week  2 times per week  3-4 times per week  5-6 times per week  1 time per day  2 or more times per day |
| 21. During the past month, not including what you just told me about (green salads, potatoes, cooked dried beans), how often did you eat **other vegetables**?  Never  1 time last month  2-3 times last month  1 time per week  2 times per week  3-4 times per week  5-6 times per week  1 time per day  2 or more times per day | 24. During the past month, how often did you have **tomato sauces** such as with spaghetti or noodles or mixed into foods such as lasagna? Do not include tomato sauce on pizza.  Never  1 time last month  2-3 times last month  1 time per week  2 times per week  3-4 times per week  5-6 times per week  1 time per day  2 or more times per day |
| 22. During the past month, how often did you have Mexican-type **salsa** made with tomato?  Never  1 time last month  2-3 times last month  1 time per week  2 times per week  3-4 times per week  5-6 times per week  1 time per day  2 or more times per day | 25. During the past month, how often did you eat any kind of **cheese**? Include cheese as a snack, cheese on burgers, sandwiches, and cheese in foods such as lasagna, quesadillas, or casseroles. Do **not** include cheese on pizza.  Never  1 time last month  2-3 times last month  1 time per week  2 times per week  3-4 times per week  5-6 times per week  1 time per day  2 or more times per day |
| 26. During the past month, how often did you eat **unpasteurized** cheese such as queso fresco, queso blanco, asadero, brie, camembert, and blue cheese?  Never  1 time last month  2-3 times last month  1 time per week  2 times per week  3-4 times per week  5-6 times per week  1 time per day  2 or more times per day | 29. During the past month, how often did you eat **whole grain bread** including toast, rolls, and in sandwiches? Whole grain breads include whole wheat, rye, oatmeal, and pumpernickel. Do **not** include while bread.  Never  1 time last month  2-3 times last month  1 time per week  2 times per week  3-4 times per week  5-6 times per week  1 time per day  2 or more times per day |
| 27. During the past month, how often did you eat **red meat**, such as beef, pork, ham, or sausage? Do **not** include chicken, turkey, or seafood. Include red meat you had in sandwiches, lasagna, stew, and other mixtures. Red meats may also include veal, lamb, and any lunch meats made with these meats.  Never  1 time last month  2-3 times last month  1 time per week  2 times per week  3-4 times per week  5-6 times per week  1 time per day  2 or more times per day | 30. During the past month, how often did you eat **chocolate** or any other types of **candy**? Do not include sugar-free candy.  Never  1 time last month  2-3 times last month  1 time per week  2 times per week  3-4 times per week  5-6 times per week  1 time per day  2 or more times per day |
| 28. During the past month, how often did you eat any **processed meat**, such as bacon, lunch meats, or hot dogs? Include processed meats you had in sandwiches, soups, pizza, casseroles, and other mixtures. Processed meats are those preserved by smoking, curing, or salting, or by the addition of preservatives. Examples are: ham, bacon, pastrami, salami, sausages, bratwursts, frankfurters, hot dogs, and spam.  Never  1 time last month  2-3 times last month  1 time per week  2 times per week  3-4 times per week  5-6 times per week  1 time per day  2 or more times per day | 31. During the past month, how often did you eat **doughnuts, sweet rolls, Danish, muffins, pan dulce, or pop-tarts**? Do **not** include sugar-free items.  Never  1 time last month  2-3 times last month  1 time per week  2 times per week  3-4 times per week  5-6 times per week  1 time per day  2 or more times per day |
| 32. During the past month, how often did you eat **cookies, cake, pie or brownies**? Do not include sugar-free kinds.  Never  1 time last month  2-3 times last month  1 time per week  2 times per week  3-4 times per week  5-6 times per week  1 time per day  2 or more times per day | 35. During the past month, how often did you eat **yogurt**?  Never  1 time last month  2-3 times last month  1 time per week  2 times per week  3-4 times per week  5-6 times per week  1 time per day  2 or more times per day |
| 33. During the past month, how often did you eat **ice cream or other frozen desserts**? Do not include sugar-free kinds.  Never  1 time last month  2-3 times last month  1 time per week  2 times per week  3-4 times per week  5-6 times per week  1 time per day  2 or more times per day |  |
| 34. During the past month, how often did you eat **popcorn**?  Never  1 time last month  2-3 times last month  1 time per week  2 times per week  3-4 times per week  5-6 times per week  1 time per day  2 or more times per day |  |

| Initials of person completing form:   \| ____ ____ ____ \| \| --- \| |  |
| --- | --- | --- |
