# Supplementary material for: The early infant gut microbiome varies in association with a maternal high-fat diet
Source: Genome Med. 2016 Aug 9;8:77. doi: 10.1186/s13073-016-0330-z (PMC4977686; doi:10.1186/s13073-016-0330-z)
Supplement: Additional file 4: — Supplemental Figures S1–S4. (PDF 296 kb) [file 13073_2016_330_MOESM4_ESM.pdf]

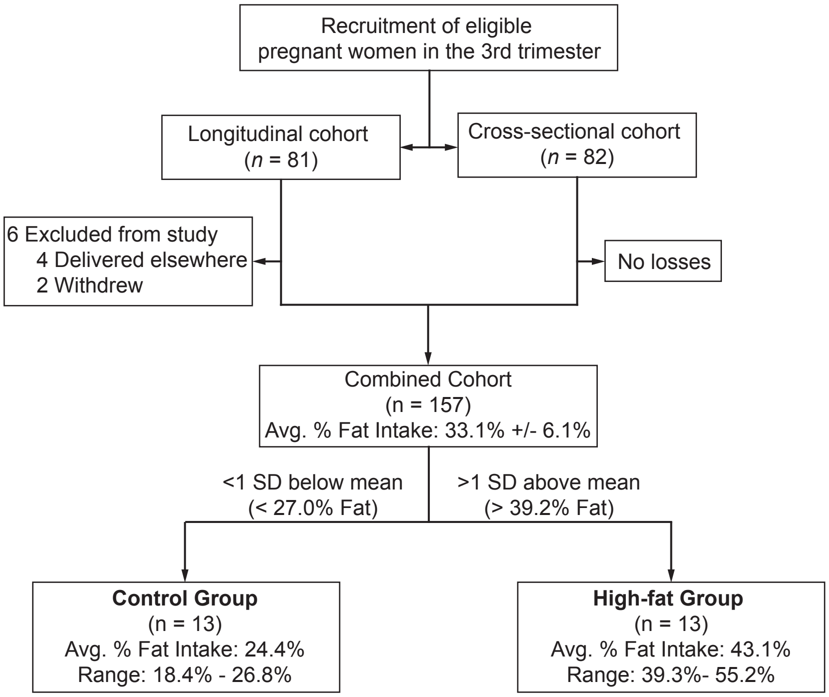

Supplemental Figure 1: Study Design Overview. Flowchart indicating the subjects enrolled in each cohort, the cohort losses, and the parameters used to selected cases (high-fat) and controls. SD = standard deviation.

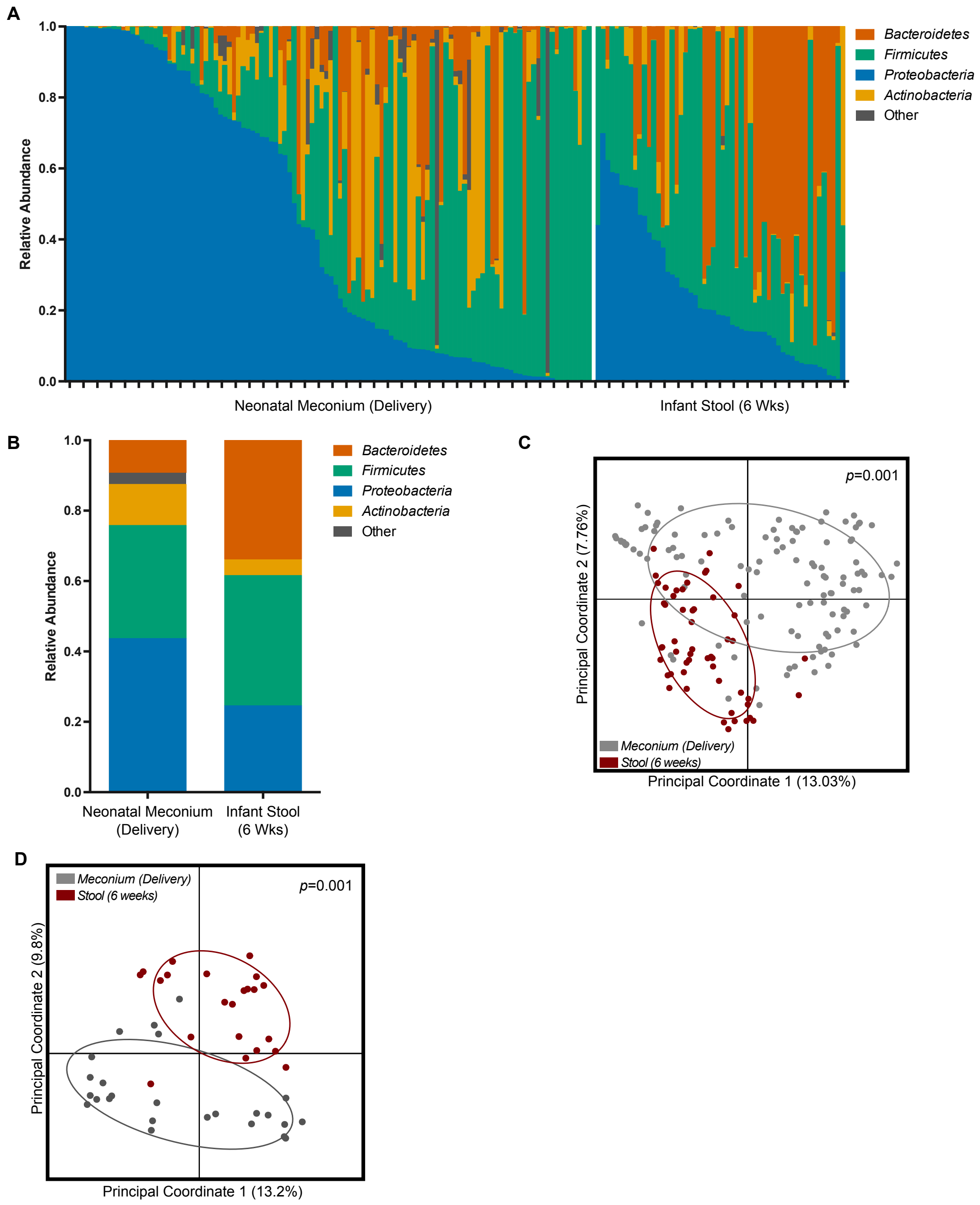

Supplemental Figure 2: The community structure of the neonatal gut microbiota at delivery and at 6 weeks differs at the phylum and OTU level. (A) Stacked-bar plots demonstrate the relative abundance of the dominant phyla found within each stool sample at delivery (left) and 6 weeks of age (right). (B) The average relative abundance of the dominant phyla found within each stool sample at delivery (left) and 6 weeks of age (right). (C) Principal Coordinate Analysis of unweighted Unifrac distances for the neonatal gut microbiota at delivery (gray) and 6 weeks (red) for all samples across the entire cohort. Cluster significance determined by PERMAVOVA (*p*=0.001). (D) Principal Coordinate Analysis of unweighted Unifrac distances for the neonatal gut microbiota at delivery (gray) and 6 weeks (red) only for samples included the maternal control or high-fat diet groups. Cluster significance determined by PERMAVOVA (*p*=0.001).

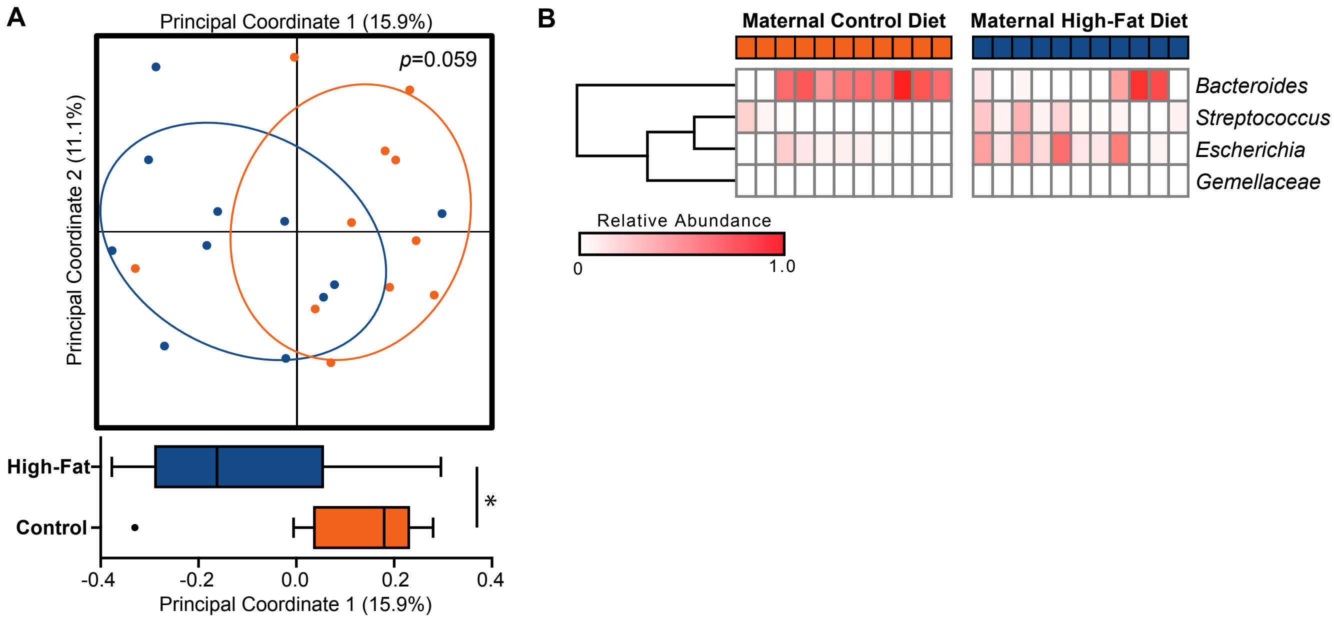

Supplemental Figure 3: The infant gut microbiota differ by virtue of maternal dietary intake of fat during pregnancy. (A) Principal Coordinate Analysis of infant stool (6 weeks) on unweighted UniFrac distances, with the distribution of the samples along the first principal coordinate axis shown below as a boxplot representing the median and interquartile range (*p=0.0192 by a Mann-Whitney U test). Cluster significance determined by PERMANOVA (p=0.059). (B) Heatmap of features that were significantly associated (p<0.05) with either a maternal high-fat or control diet during pregnancy.

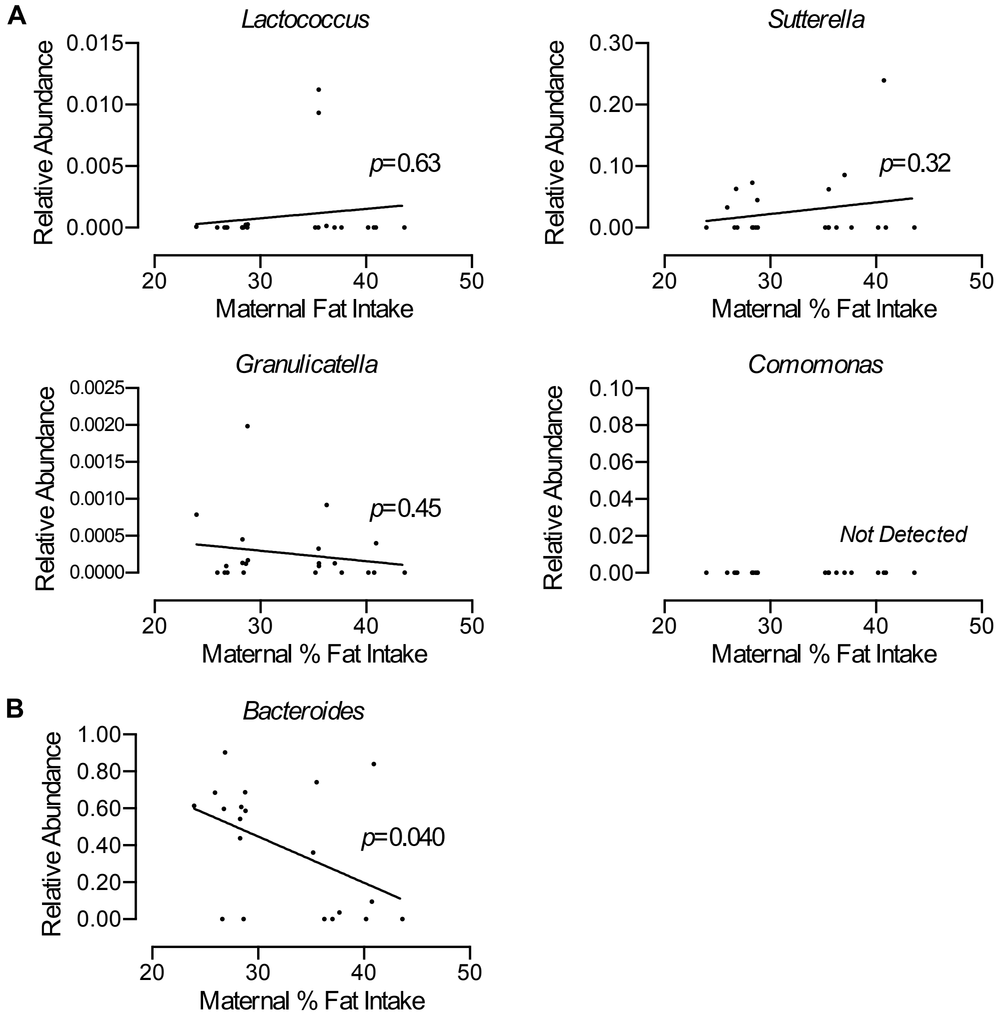

Supplemental Figure 4: Correlations between maternal fat intake and taxa abundance within the infant stool at 6 weeks of age. (A) Each taxa was previously identified by LEfSe to be significantly different between the maternal high-fat and control groups at the time of delivery. However, no significant correlation between maternal fat intake during gestation and taxa abundance was seen for these taxa at 6 weeks of age (all $p>0.05$). (B) Even when exclusively formula fed infants were removed from analysis, a significant correlation between the relative abundance of *Bacteroides* and maternal fat intake during gestation remained ($p=0.04$).
